# 2624. The Usual Suspects: A Retrospective Analysis of Pediatric Respiratory Viral Testing During the Pandemic

**DOI:** 10.1093/ofid/ofad500.2237

**Published:** 2023-11-27

**Authors:** Iris Cazali, Diego Erdmenger, Karin roldan

**Affiliations:** Hospital Roosevelt, Guatemala, Sacatepequez, Guatemala; Hospital General San Juan de Dios, Guatemala, Guatemala, Guatemala; Hospital Roosevelt, Guatemala, Sacatepequez, Guatemala

## Abstract

**Background:**

Contention measures implemented during the pandemic drastically changed the epidemiology of viral respiratory infections. Some reports have shown a sharp decrease in Emergency department visits for respiratory illnesses in the first months of the pandemic with an increase in Respiratory Syncytial Virus (RSV) and Enterovirus (EV) in subsequent months.

The concern caused by COVID-19 during the beginning of the pandemic may have led healthcare professionals to underdiagnose other respiratory viral infections. The aim of this study is to evaluate the behavior of viral infection through the pandemic.

**Methods:**

A retrospective analysis was conducted to determine temporal changes in respiratory panel use and viral epidemiology in the pediatric ED from 2018 to 2022. Medical records were used to determine ICU admission and mortality in those with a positive respiratory panel.

**Results:**

During the study period, a total of 10,000 respiratory PCR panels were performed in the pediatric ED. There was a significant increase in mean number of panels performed per year during the pandemic period compared to two years prior (2899 vs 198 panels/year). However, the Positivity rate decreased considerably during the pandemic (38.27% vs. 63.54%).

Of the 10,000 panels performed, COVID-19 was identified in in 993 cases (9.93%), while other respiratory viruses were identified in 31.27% of cases. The most prevalent virus identified were EV (n=1411, 14.9%) and RSV (n=483, 5.1%). These viruses also had the highest rate of ICU admission and mortality.

Patients that tested positive for COVID-19 had a lower rate of ICU admission compared to those infected by other viruses (RR=0.88; CI95%=0.78-0.99). Additionally, the mortality rate associated with COVID-19 was significantly lower than that of other viruses (RR=0.62; CI95%=0.49-0.79).

PCR Panel Positivity Rate and Viral Infections in Pediatrics ED from 2018-2022

**Figure:**
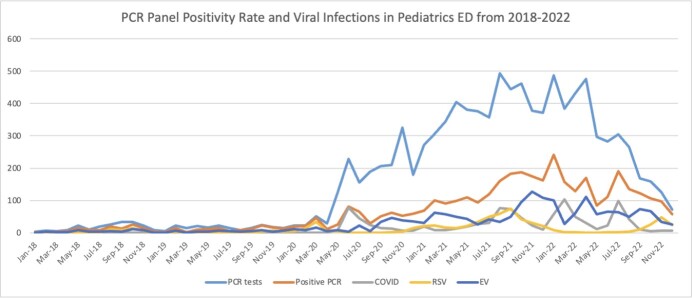

PCR viral panel tests performed, positive tests, and viral isolates from 2018 to 2022

**Conclusion:**

The pandemic increased the use of PCR testing for respiratory illnesses, providing a better picture of seasonal epidemiology of viral infections. Although COVID-19 infection in children was a major of concern, this report shows that throughout the pandemic other viruses were more prevalent and significantly more severe. This study serves as a reminder than even in times of emerging infections, healthcare professionals shouldn’t forget the usual causes of infection.

**Disclosures:**

**All Authors**: No reported disclosures

